# Association Between Diet Quality Linked to Gut Microbiota and Cardiovascular Risk Among South Asian Adults: A Cross-Sectional Study

**DOI:** 10.7759/cureus.88395

**Published:** 2025-07-20

**Authors:** Ayesha Ghazal Jamali, Aimen Ilyas, Faisal Wali Ahmed, Ayesha Mansoor, Syeda Zainab Hassan, Shivam Singla, Bhavna Singla, Khaled Kettaneh, Raida Jannath Mim, Mavia Habib, Ahmed A Iqbal

**Affiliations:** 1 Medicine and Surgery, Liaquat University of Medical and Health Sciences, Jamshoro, PAK; 2 Medicine, Faisalabad Medical University, Faisalabad, PAK; 3 Critical Care Medicine, King Saud Medical City, Riyadh, SAU; 4 Special Pathology, Community Medicine, ENT, Eye, Faisalabad Medical University, Faisalabad, PAK; 5 Special Pathology, Community Medicine, ENT, Eye, Allied Hospital Faisalabad, Faisalabad, PAK; 6 Internal Medicine, Jinnah Hospital, Lahore, PAK; 7 Internal Medicine, TidalHealth Penninsula Regional, Salisbury, USA; 8 Internal Medicine, Erie County Medical Center Hospital, Buffalo, USA; 9 Cardiology, Mutah University, Kerak, JOR; 10 Internal Medicine, Sylhet Women's Medical College, Sylhet, BGD; 11 Internal Medicine, Services Hospital Lahore, Lahore, PAK; 12 Medicine, NeuroWave Research Center, Islamabad, PAK

**Keywords:** cardiovascular risk, dietary patterns, diet quality, gut microbiota, interheart score, south asian adults

## Abstract

Background

Cardiovascular disease (CVD) is a significant public health issue worldwide that depends on various lifestyle and metabolic factors. Emerging research suggests that dietary patterns affecting the gut microbiota may influence cardiovascular risk. Nevertheless, not much research has been conducted on this relationship within South Asian communities. This study investigates the relationship between diet quality, as defined by patterns associated with gut microbiota in prior literature, and modifiable cardiovascular risk factors among South Asian adults.

Methods

This cross-sectional study was conducted from January to May 2025 in Pakistani community clinics and health centres. The study employed convenience sampling and recruited 385 South Asian adults. The participants were questioned using a structured questionnaire, and their demographic data, Global Diet Quality Questionnaire (GDQQ, Pakistan version), and INTERHEART Modifiable Risk Score (IHMRS) were obtained. Although gut microbiota was not directly assessed, diet quality was inferred to be linked with microbial changes based on previously validated dietary patterns in microbiome research. Both GDQQ and IHMRS have been validated in South Asian populations, with IHMRS demonstrating strong predictive performance (AUC > 0.75). Age categories used in the analysis included six groups: 18-24, 25-34, 35-44, 45-54, 55-64, and 65 years and older. The data were analysed using SPSS v.26, and associations between diet quality and cardiovascular risk were assessed using descriptive statistics, t-tests, analysis of variance (ANOVA), Pearson's correlation, and multivariable linear regression controlling for age, gender, physical activity, and comorbidities.

Results

Females had a significantly higher mean GDQQ score (M = 49.94, SD = 6.23) than males (M = 48.03, SD = 7.89), p < 0.001. Diet quality was inversely associated with cardiovascular risk (r = -0.827, p < 0.001). Multivariable regression confirmed that higher GDQQ scores were a strong independent predictor of lower IHMRS (β = -0.827, p < 0.001) after adjusting for key confounding variables. Significant relationships were also found between age and self-reported physical activity, as well as dietary quality and risk scores, using appropriate tests (t-tests, ANOVA, Pearson correlation). However, the use of convenience sampling may introduce selection bias and limit the generalizability of the results.

Conclusion

This study demonstrates an inverse correlation between diet quality, which is linked to gut microbiota based on previous evidence, and cardiovascular risk among South Asian adults. Older people had better diet quality, which was associated with reduced risk, whereas younger adults had a higher chance of poor diet and higher risks. These findings support the potential for culturally tailored dietary interventions targeting microbiome-related pathways to reduce the CVD burden in this region. However, the gut microbiota link is inferred, and further research involving direct microbiome assessment is warranted.

## Introduction

Cardiovascular disease is the most significant cause of death in the USA and is responsible for more than 39% of all deaths [1]. The risk of developing cardiovascular disease over time varies significantly based on personal risk factors. Individuals with few risk factors are considerably less likely to experience heart-related problems or pass away compared to those with multiple risk factors, so learning how to manage risks is essential throughout one's life [2].

Noncommunicable diseases are responsible for more global deaths than any other condition, and heart diseases cause most of these deaths [3]. Having insulin resistance in diabetes increases the likelihood of cardiovascular disease. Among the main risk factors are obesity, having too much cholesterol, and high blood pressure [3]. The number of cardiovascular disease cases is declining among rich populations; however, rates are staying the same or are still rising among the poor [4]. Therefore, there is a need for strategies and policies that address health issues resulting from income disparities [4].

Metabolism, immune balance, and immunity to infection are supported by the human gut microbiota, which is influenced by birth methods, diet, early environmental factors, and the use of antibiotics. Discoveries in genomics have made it clearer how dysbiosis may contribute to several diseases, highlighting the importance of focusing more on host-microbe and gut health studies [5,6]. A healthy gut microbiota is essential for our health, but if its balance is disrupted (dysbiosis), it contributes to cardiovascular disease by increasing inflammation and producing harmful microbial byproducts. In this way, we can develop new gut therapies to fight against CVD [7]. The gut microbiota is associated with heart disease, and recent studies have identified specific microbial components, such as trimethylamine N-oxide and phenylacetylglutamine, as key contributors to its development [8]. Detecting these compounds is crucial for maintaining heart health, as they interact with the body through specific receptors, suggesting the potential development of new microbial therapies for cardiovascular diseases [8].

Diet has a significant impact on the gut microbiota, influencing the composition of bacteria and the metabolites they produce [9]. They have a substantial effect on human health. Researchers are beginning to investigate how comprehensive nutrition and diet plans may benefit the gut microbiota and overall health [9]. The type of food people eat can alter their gut microbes, which in turn affects their health. Eating unbalanced food can upset the gut flora, increasing the risk of disease, whereas eating well-balanced meals encourages the growth of beneficial gut bacteria [10]. Consuming a high amount of fibre from plants promotes the development of healthy bacteria in the gut, resulting in the production of short-chain fatty acids that help protect against heart disease [11]. Utilizing nutrition to modulate the gut microbiome may help control chronic diseases and lead to more effective treatment options [11].

Diversity in South Asian dietary patterns is influenced by cultural, regional, and socioeconomic factors, which can impact gut microbiota and cardiovascular health. However, little research has been conducted on how changes in the gut microbiome occur with different dietary patterns and their relationship to heart health in this group. Since cardiovascular diseases are prevalent and eating habits are diverse in South Asia, understanding their impact on the gut microbiota is crucial at this time. In this study, gut microbiota composition was not directly assessed; instead, it was inferred from dietary patterns previously linked to microbial changes in existing literature. In this study, "South Asian adults" refers specifically to adults residing in Pakistan.

This study employs a cross-sectional design to investigate the association between diet quality and cardiovascular risk among South Asian adults. The study considers diet quality as a proxy for gut microbiota activity based on previously validated dietary patterns but does not include any direct microbial measurements.

Traditional risk factors alone cannot fully explain the high rate of cardiovascular disease in South Asians. Studies are showing that the heart benefits from gut microbiota, as it controls the dietary effects on metabolism and inflammation. However, there is not much information about the impact of the South Asian diet on the microbes in the intestines and heart health.

Since people in South Asia have diverse cultures and consume a variety of foods, it is essential to understand the impact of diet and gut bacteria on cardiovascular health. This study adjusts for traditional cardiovascular risk factors, including hypertension and diabetes, in its analysis.

This study aims to investigate the relationship between dietary patterns associated with gut microbiota and modifiable cardiovascular risk factors in South Asian adults. To narrow the focus, the study specifically examines the relationship between diet quality and cardiovascular risk, using gut microbiota-linked dietary patterns as an indirect indicator rather than aiming to assess microbial indicators directly.

Primary objectives

The primary objective of this study was to investigate the association between cardiovascular risk and dietary patterns that have been previously linked to gut microbiota in South Asian adults and to identify the primary eating habits of this population and explore how these dietary behaviors relate to cardiovascular risk.

Secondary objectives

Secondary objectives included assessing whether dietary quality (measured using validated questionnaires) and interpreted based on literature linking it to microbial activity, is associated with cardiovascular risk, as well as determining which dietary factors (measured using validated questionnaires) can affect gut-heart interactions and lead to the formulation of a culturally specific preventive diet among South Asian populations.

## Materials and methods

Study design and methods

The study employed a cross-sectional design to investigate the relationship between diets associated with gut bacteria and heart health in adults from South Asia. This design was chosen due to its feasibility and capacity to provide a snapshot of the associations between diet and cardiovascular risk within a resource- and time-restricted environment. Although it cannot be used to infer causality, it enables population-based analysis and hypothesis testing. To represent different parts of society, individuals were enrolled in community health centres, outpatient clinics, and general medical practices in many areas of Pakistan. This approach enabled consideration of individuals with a diverse range of lifestyles and eating habits.

Data was collected using designed questionnaires that the interviewers administered. Some of the sections on the questionnaire covered diet, daily routines, health status, and known problems such as blood pressure and diabetes. Participants completed a food frequency questionnaire specifically designed for South Asians to reflect their typical dietary patterns. Gut microbiota composition was not directly measured in this study; instead, it was approximated based on dietary patterns that had been related to changes in microbes in other literature. Each participant chose to participate and gave their permission before any research data were taken. Because of this design, researchers could observe how eating certain foods contributes to heart problems, laying the groundwork for future studies exploring the relationship between diet and gut health in people from South Asia.

Sample size and technique

It is unknown how many adults in South Asia develop heart disease by eating a certain diet, so this study worked with an infinite population. The equation for calculating the sample size was used:

\[n = \frac{Z^2 \cdot p (1 - p)}{d^2}\]

Here, Z equals the standard needed for the confidence level, p indicates the predicted proportion learned from other studies, and d indicates the acceptable error being investigated. The value of Z was established as 1.96, which means a 95% confidence level was used. It was also determined that the margin of error (d) was 0.05. To match the findings of other research that estimated 50% of dietary risk among South Asians, it was decided to set p as 0.50 to obtain the correct sample size [12].

To ensure the validity of this research, the minimum sample size for the study was determined to be 385 participants. The survey targeted a larger number of people than usual because some were unwilling to participate, and there was missing data. Participants were recruited for the study through convenience sampling at community gatherings, local screening events, and various clinics. While this approach facilitated recruitment, it may introduce selection bias, which limits the generalizability of the findings to the broader population. Using this method, researchers only included participants who qualified and were active at the time of data collection.

Inclusion criteria

This study involved South Asian adults who were at least 18 years old and able to provide informed consent and answer all the questions. Participants were selected if they had not undergone any major surgery on the digestive tract or any primary infection that could affect the function of their gastrointestinal tract. Participants included in the study had to demonstrate normal cognitive skills to understand and answer the questions accurately.

Exclusion criteria

Participants were excluded from the study if they had taken antibiotics, probiotics, or other medications that could significantly alter the gut microbiome within the past four weeks. Since the dietary requirements and metabolism differ for pregnant and lactating women, these individuals were excluded from the study. For this reason, participants with inflammatory bowel disease or celiac disease were excluded from the analysis to avoid potential issues related to gut health.

Data collection tools

We used a structured questionnaire with three main parts: questions about the participants' demographic information, diet, and cardiovascular risk. The tools used in the questionnaire include both trusted standards and questions made by the researchers to cover all the essential and relevant aspects.

Demographic information

In the first part, researchers gathered data to investigate whether cardiovascular risk is associated with personal or social factors. This required gathering information on a person's age, gender, marital status, educational background, employment history, and various habits, such as smoking and exercise habits. Due to these factors, it became clear where to look for differences in the study participants' diets and health.

Global Diet Quality Questionnaire (GDQQ - Pakistan version)

The second part included the Global Diet Quality Questionnaire (GDQQ), which was developed for Pakistan by the Global Diet Quality Project, an initiative of the International Food Policy Research Institute (IFPRI)'s intake group. Launched in 2019, the DQQ is designed to assess the health and diversity of diets among people living in various countries. Pakistan's food categories typically highlight the meals people usually prepare, including roti, rice, pulses, dairy products, traditional sweets, fried snacks, and various drinks. The dietary intake of 29 different food components is assessed to evaluate the potential benefits and risks associated with them. In this study, participants were asked whether they had consumed each of the 29 food groups in the past 24 hours. Responses were coded as “yes” (1) or “no” (0), and total scores were calculated by summing the number of food groups consumed. Higher scores indicated better dietary diversity and quality. Although this tool was adapted for Pakistan, further psychometric validation in this specific population was not conducted as part of this study [13].

INTERHEART Modifiable Risk Score (IHMRS)

For this study, we relied on the INTERHEART Modifiable Risk Score introduced and validated in 2010 by exploring the original INTERHEART study from 2004 by Yusuf et al. The tool measures a person’s chance of cardiovascular problems by studying factors that can be managed, like smoking, high blood pressure, diabetes, weight, diet habits, physical exercise, stress due to lifestyle, and how much alcohol they drink. It has been reported that the INTERHEART score performs well in several populations, such as South Asians, as its area under the receiver operating characteristic curve (AUC) is frequently greater than 0.75, indicating intense discrimination of risk between individuals. Since it is reliable and straightforward, it becomes a valuable risk assessment tool for people in Pakistan [14].

Procedure

Individuals for the study were recruited from various community centres and clinics, provided they had signed the consent forms. The study’s data collection took place from January 2025 to May 2025. Participants took part in the survey by either answering questions themselves or with assistance from interviewers, depending on their preference. The questionnaire was primarily self-administered. However, every data collector was trained according to a standardised protocol to ensure the consistent administration of the survey and minimise the extent of inter-observer variability. Trained data collectors assisted when participants had difficulty understanding or reading the items. All personal data that could identify a patient was removed from the study information. As a result, data were collected in an ethical, respectful, and accurate manner, including individuals from diverse cultural and socioeconomic backgrounds.

Statistical analysis

Data analysis was conducted in IBM SPSS Statistics version 26 (IBM Corp., Armonk, NY). The demographic and clinical characteristics of participants were summarised using means, standard deviations, frequencies, and percentages. To determine the normality of the data, Q-Q plots were created for the DQQ and IHMRS, and the normality was found to be approximately normal. While scatterplots and boxplots were not formally designed to evaluate linearity and outliers, the visual distribution of values and the high significance in correlation results suggest that the assumptions for Pearson correlation were likely met. This is acknowledged as a limitation in the current analysis. To answer this question, a Pearson correlation analysis was conducted to examine the relationship between DQQ and IHMRS scores. Independent samples t-tests were used to compare the groups based on gender, and one-way analysis of variance (ANOVA) was applied to assess differences between age categories and levels of physical activity. The Levene test did not statistically test homogeneity of variances; it was assumed. This limitation should be taken into account when interpreting the results. A multiple linear regression analysis was performed to identify predictors of INTERHEART Modifiable Risk Scores, with diet quality, age, gender, physical activity (frequency and self-rated level), and medical conditions entered as independent variables. Additionally, the chi-square test was used to analyse the relationships between demographic variables (including age) and the frequency of reported physical activity and existing medical conditions. Age was categorised into six groups (18-24, 25-34, 35-44, 45-54, 55-64, and ≥65) to facilitate the proper application of chi-square tests. All analyses were conducted with the least notable limitations of p < 0.05, and any interconnections between dietary quality, cardiovascular risk factors, and demographic or behavioural conditions were identified. Missing data were handled using listwise deletion, and only complete cases were included in each analysis.

Ethical considerations

The research was conducted in accordance with the ethical guidelines established for studies involving human subjects. IRB-2025-0032 of the Neurowave Research Centre, Islamabad, approved and reviewed the study protocol before the commencement of the research. This approval ensured that ethics were carefully followed by respecting people, providing benefits, and maintaining confidentiality.

All participants in the study received sufficient information about its objectives, methodology, potential risks, and benefits. Before being involved, every participant signed a written informed consent form. Taking part in the research was purely at each individual’s discretion, so no one was pressured to join, and anyone could opt out at any moment without consequences. The IRB approved data security measures and prescribed that participant information be treated as confidential. Identifiable data was stored on secure, password-controlled electronic devices and in locked filing stores or paper files. Access was restricted to the authorized members of the research team.

## Results

Table 1 demonstrates demographic data of participants of the study (N = 385). The most numerous age group was 35-44 years (n = 116, 30), then 45-54 years (n = 97, 25), and 25-34 years (n = 79, 20). The number of males (n = 204, 53%) was bigger than that of females (n = 181, 47%). Most of them were married (n = 165, 43%); others were divorced (n = 93, 24%), single (n = 91, 24%), or widowed (n = 36, 9%). On education, the largest percentage attended primary school (n = 117, 30%), secondary school (n = 109, 28%), and no formal education (n = 72, 19%). Regarding employment, most were unemployed (n = 133, 34%), followed by large percentages of students (n = 95, 25%) and employed people (n = 77, 20%). Regarding physical activity, most of the participants stated that they are physically active 2-3 times a week (n = 121, 31%) or every day (n = 114, 30%), and evaluating their level of activity, mentioned that it is moderate (n = 149, 39%) or high (n = 100, 26%). The most prevalent health conditions were hypertension (n = 154, 40%), diabetes (n = 107, 28%), and high cholesterol (n = 85, 22%), and a few did not report any of these conditions (n = 9, 2%). Of the 410 approached individuals, 385 provided a complete response to all key variables, including GDQQ and IHMRS. Incomplete and /or missing responses (n = 25) were eliminated through listwise deletion. Therefore, all statistical modeling, including correlations and regressions, was conducted on the final complete sample (N = 385). No imputation process was used.

**Table 1:** Demographic characteristics of participants (N=385) f: frequency; %: percentage.

Variable	f	%
Age	-	-
18-24 years	41	10
25-34 years	79	20
35-44 years	116	30
45-54 years	97	25
55-64 years	42	11
65 years or above	10	4
Gender	-	-
Male	204	53
Female	181	47
Marital status	-	-
Single	91	24
Married	165	43
Divorced	93	24
Widowed	36	9
Educational level	-	-
No formal education	72	19
Primary school	117	30
Secondary school	109	28
College/University (Undergraduate)	56	14
Postgraduate or higher	31	8
Employment status	-	-
Student	95	25
Employed	77	20
Homemaker	30	8
Unemployed	133	34
Retired	50	13
How often do you engage in physical activity (e.g., walking, exercise, sports)?	-	-
Daily	114	30
2-3 times per week	121	31
Once a week	79	20
Rarely	38	10
Never	33	8
How would you rate your physical activity level?	-	-
High	100	26
Moderate	149	39
Low	78	20
Very low	58	15
Do you currently have any of the following conditions?	-	-
Diabetes	107	28
Hypertension	154	40
High cholesterol	85	22
Obesity	30	8
None of the above	9	2

**Figure 1:**
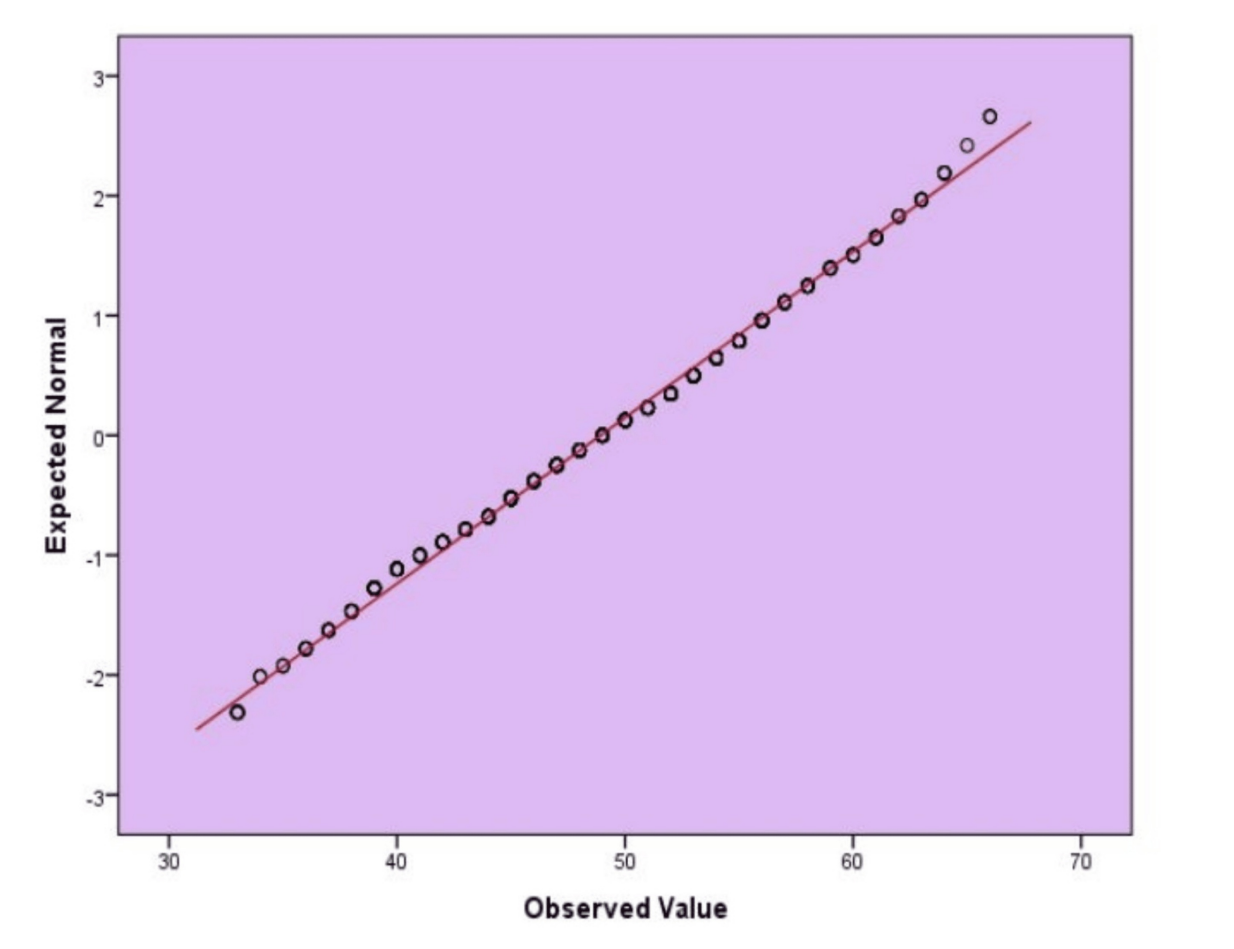
Standard Q-Q plot assessing the distribution of Diet Quality Questionnaire (DQQ) scores

Figure 1 displays a plot of the Q-Q of DQQ scores. The points are near the diagonal line, suggesting that they are normally distributed with the scores. There are some indications of slight skewness (small deviations at the extremes). Still, the test of the assumption of normal distribution is otherwise satisfied reasonably adequately to use the usual parametric tests.

**Figure 2:**
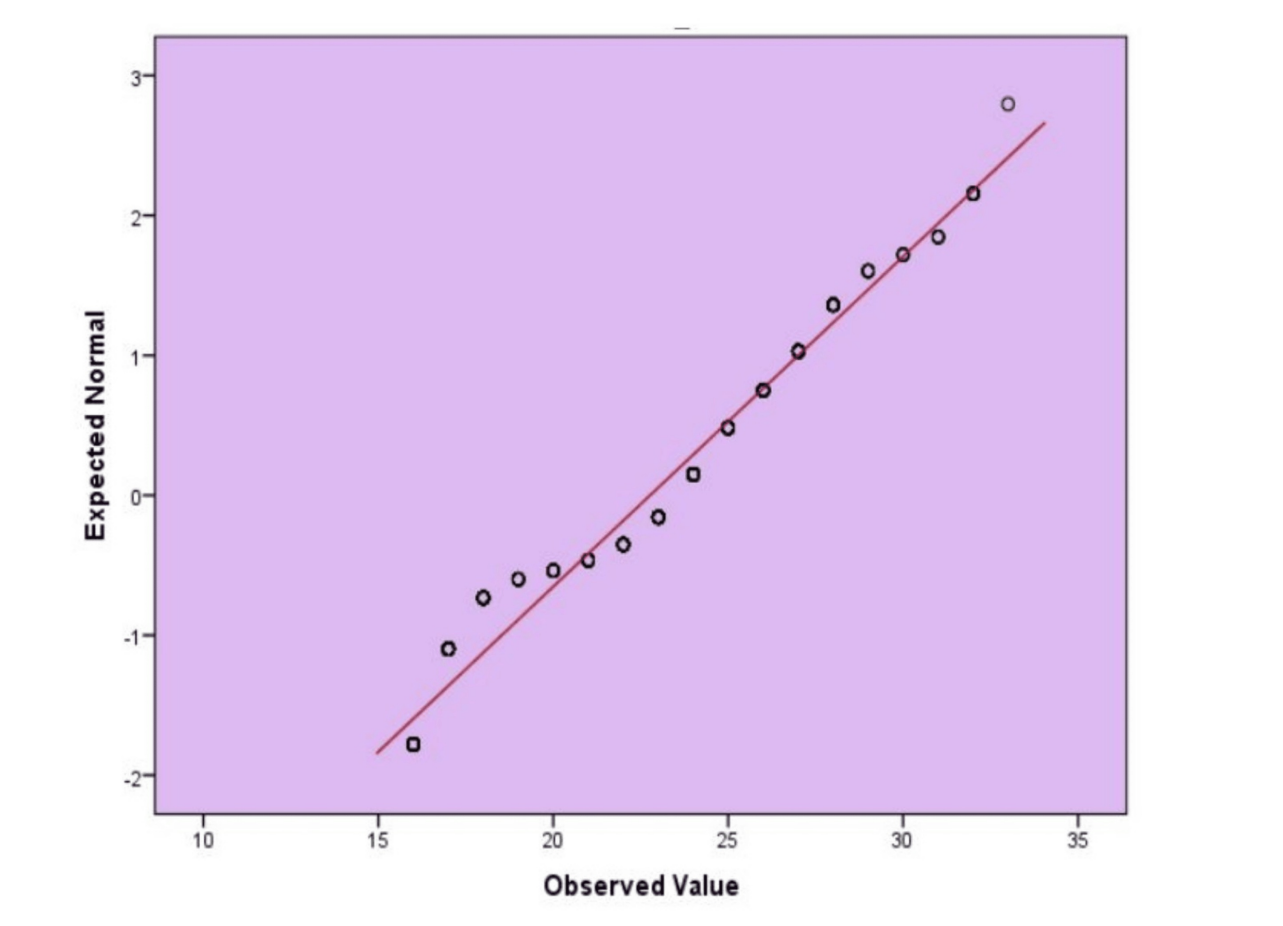
Standard Q-Q plot assessing the distribution of INTERHEART Modifiable Risk Scores (IHMRS)

Figure 2 presents a normal Q-Q plot of IHMRS. The data points are drawn near the diagonal line, which shows that the distribution of the scores is normal to a large extent. This justifies the fact that statistical procedures of assuming normality are correct.

Table 2 reports the intercorrelation between the DQQ and the IHMRS. The findings show that the two variables are negatively correlated with each other, and the correlation is strong and statistically significant (r = -0.827, p < 0.001), implying that the individuals with high diet quality scores are more likely to be individuals with lower scores of the cardiovascular risk modifiable scores. This implies that with improved eating habits, the risk of contracting cardiovascular disease is minimized. The inverse correlation shows the protective effect of a healthy diet in the mitigation of potentially changeable risk factors, which include blood pressure, level of cholesterol, and lifestyle practices such as lifestyle captured by the INTERHEART score. A significant value of this association is high, and it cements the strength of this association among the population represented by the study.

**Table 2:** Intercorrelation between study variable **: p < 0.001 considered significant; correlation: Pearson correlation.

Variable	Diet Quality Questionnaire	INTERHEART Modifiable Risk Score	p
Diet Quality Questionnaire	-	-0.827	<0.001^**^
INTERHEART Modifiable Risk Score	-0.827	-	<0.001^**^

Table 3 includes gender comparisons of diet quality and cardiovascular risk scores. The results reveal that females scored on average higher on diet quality (M = 49.94, SD = 6.23) than did males (M = 48.03, SD = 7.89); this difference was statistically significant (t = -2.611, p < 0.001), and minor to moderate in magnitude (Cohen's d = 0.27). The 95% confidence interval for the mean difference in GDQQ scores ranged from -3.35 to -0.47, indicating a precise estimate of the effect. This implies that the women who participated in the study adopted healthier eating habits. Conversely, there was no statistically significant distinction in the IHMRS between males (M = 22.64, SD = 4.44) and females (M = 22.91, SD = 4.00), t = -0.609, p = 0.543, and the small effect size (Cohen d = 0.06). The findings indicate that there is a gender variation in the quality of diet; however, the level of modifiable CVD risks was comparable in the male and female samples.

**Table 3:** Comparison among variables (gender) M: mean; SD: standard deviation; LL: lower limit; UL: upper limit; Cl: confidence interval; Statistical test: independent samples t-test; * rho < 0.001 considered significant.

Variable	Male (N=204); M±S.D	Female (N=181); M±S.D	t	p	Cl 95% LL	UL	Cohen’s D
Diet Quality Questionnaire	48.03±7.89	49.94±6.23	-2.611	<0.001^**^	-3.348	-0.472	0.27
INTERHEART Modifiable Risk Score	22.64±4.44	22.91±4.00	-0.609	0.543	-1.115	0.588	0.06

Table 4 shows the comparison of diet quality and cardiovascular risk scores with inclination by age group (six groups) as measured by one-way ANOVA. Age was found to have an important influence on the quality of the diet, F (5, 379) = 10.52, p < 0.001, and an average effect size (632 = 0.122). The socio-demographic variables have also remained the same, with a steady increase in the scores of diet quality across the various ages, with the poorest scores being recorded by the 18-24 age bracket (M = 45.83, SD = 6.37) and the best scores being registered at the age 65 years and above (M = 55.00, SD = 3.68). The pattern indicates that the sample of older individuals practised healthier eating patterns than younger participants. There was also a significant but small effect size difference in IHMRS by age group, F (5, 379) = 4.19, p = 0.011, (eta-squared = 0.052). The cardiovascular risk scores also dropped as age advanced, with the lowest score being expressed in the oldest group (M = 20.54, SD = 4.11) and the highest in the group of younger individuals (18 24 years; M = 25.00, SD = 1.94). This shows that younger people in this sample could have a higher modifiable cardiovascular risk, although they have more opportunities to avoid developing long-term health problems. Although the assumptions of normality were checked, no formal test for homogeneity of variances was conducted using the Levene test. No post hoc analyses of age groups were done, so the interpretations of group differences must be cautious and based on the mean level trends.

**Table 4:** Comparison of variables (age) M: mean; SD: standard deviation; F: ratio (variance between groups/within groups); r ^ 2: effect size; Statistical test: one-way analysis of variance (ANOVA); * p < 0.05  ** p < 0.01 considered significant.

Variable	18-24 years (N=41); M±S.D	25-34 years (N=79); M±S.D	35-44 years (N=116); M±S.D	45-54 years (N=97); M±S.D	55-64 years (N=42); M±S.D	65 years or above (N=10); M±S.D	p	F (5,379)	η2
Diet Quality Questionnaire	45.83±6.37	46.80±7.28	47.91±6.96	51.88±7.13	50.50±5.99	55.00±3.68	<0.001^**^	10.52	0.122
INTERHEART Modifiable Risk Score	25.00±1.94	23.70±4.40	22.36±4.27	22.11±4.03	21.67±4.12	20.54±4.11	0.011^*^	4.19	0.0052

Table 5 indicates that the quality of the diet and cardiovascular risk scores were significantly associated with the level of physical activity as self-reported. Amazingly, the least frequent and non-exercisers had the same quality diet scores as the less or never exercisers, with a significant difference in the scores of each of the categories as compared to the less or never exerciser, F (4, 380) = 18.90, p < 0.001, and 0.166. A near-comparable pattern was evident in IHMRS, which marked the highest scores of IHMRS in the group that exercised daily (M = 24.39), followed by the minimal worth of IHMRS in the seldom exercisers (M = 21.83), F (4, 380) = 4.26, p = 0.002, 0.043. Such results are counterintuitive and can be related to the compensatory behaviour of the diet self-reporting bias or other confounding factors, including age, health, or comorbid diseases, and therefore, they should be explored in further research.

**Table 5:** Comparison of variables (self-related physical activity) M: mean; SD: standard deviation; F: F-ratio (variance between groups/within groups); n²: effect size; Statistical test: one-way analysis of variance (ANOVA); ** p < 0.01 considered significant.

Variable	daily (N=114); M±S.D	2-3 times per week (N=121); M±S.D	Once in a week (N=79); M±S.D	Rarely (N=38); M±S.D	Never (N=33); M±S.D	p	F (4,380)	η2
Diet Quality Questionnaire	46.07±6.89	47.52±6.99	51.27±6.59	52.24±6.63	54.55±4.68	<0.001^**^	18.90	0.166
INTERHEART Modifiable Risk Score	24.39±3.14	23.37±3.93	23.70±4.63	21.83±4.02	22.45±4.32	0.002^**^	4.26	0.043

Table 6 presents a multivariable linear regression analysis that forecasts INTERHEART Modifiable Risk Scores (IHMRS) based on diet quality, age, gender, physical activity (frequency and self-rated degree), and the presence of medical conditions. The overall model was significant and accounted for a high percentage of the variance in risk scores (R² = 0.782, Adjusted R² = 0.776). The strongest independent predictor was diet quality, with higher scores being strongly related to a reduced cardiovascular risk (lowest: 0.827, p < 0.001). There was also a strong inverse correlation between age (β = -0.078, p = 0.019), indicating that older participants had a lower modifiable risk of disease. The female gender had a minimally decreased risk score (beta = -0.063, p = 0.004). Interestingly, the frequency of physical activity was associated with a greater cardiovascular risk (beta = 0.115, p < 0.001). In contrast, a higher self-rated physical activity level was associated with a reduced risk (beta = -0.085, p = 0.002), suggesting perhaps a misalignment between self-reported and actual physical activity levels. Also, having medical conditions, including hypertension or diabetes, caused a high level of cardiovascular risk (0.247, p < 0.001). Overall, the results suggest that controlling one's diet and managing chronic health conditions play a significant role in mitigating cardiovascular threats while also highlighting the confounding effects of physical activity on heart health.

**Table 6:** Multivariable linear regression predicting INTERHEART Modifiable Risk Scores from diet quality, demographics, physical activity, and medical conditions B: coefficient; S.E: standard error; β: standardized coefficient; LL: lower limit; UL: upper limit; Cl: confidence interval; *: p < 0.05 considered significant; **: p < 0.01 considered significant.

Variable	B	95% Cl LL	UL	S.E	β	P
Model summary	-	-	-	-	-	-
Constant	19.885	16.186	23.58	1.881	-	< 0.001^**^
Diet Quality Questionnaire	-0.942	-1.024	-0.860	0.042	-0.827	< 0.001^**^
Age	-0.265	-0.486	-0.044	0.112	-0.078	0.019^*^
Gender	-0.540	-0.902	-0.177	0.185	-0.063	0.004^**^
Frequency of Physical Activity	0.420	0.221	0.619	0.101	0.115	<0.001^**^
Self-Rated Physical Activity Level	-0.360	-0.585	-0.135	0.115	-0.085	0.002^**^
Presence of Medical Conditions (e.g., diabetes, hypertension, etc.)	1.050	0.771	1.328	0.142	0.247	<0.001^**^
Model R²	0.782	-	-	-	-	-
Adjusted R²	0.776	-	-	-	-	-

Table 7 illustrates the prevalence of physical activity and medical conditions across various age groups. The relationship between age and self-rated frequency of physical activity was found to be statistically significant (p < 0.018, χ² = 35.4), indicating that the distribution of physical activity differed meaningfully by age. For example, the prevalence of daily physical activity was highest among participants aged 18-24 (n = 23) and 35-44 years (n = 38), whereas less physical activity was more typical of older age groups. Similarly, a significant correlation was found between age and active health problems (p = 0.002, r = 0.43). Those aged between 35 and 54 years showed high rates of hypertension and high cholesterol levels, and the young population (18-24 years) had comparatively lower rates of medical conditions. These findings suggest that physical activity and the prevalence of chronic conditions are closely related to age. Age and other demographic variables were categorized to ensure appropriate cell sizes. Based on the sample distribution, expected frequencies were likely sufficient for chi-square assumptions (i.e., ≥ 5 in at least 80% of cells). No cells with a count of zero were observed.

**Table 7:** Descriptive statistics of demographic variables (age, self-related physical activity, current medical conditions) f: frequency; %: percentage; p: level of significance; p-values calculated using the chi-square test; *: p < 0.05 considered significant; **: p < 0.01 considered significant.

Variables	f	Daily	Self-related physical activity 2-3 times per week	Once a week	Rarely	Never	p	x^2^	Diabetes	Current medical conditions Hypertension	High cholesterol	Obesity	None	p	x^2^
Age	-	-	-	-	-	-	<0.018^*^	35.4	-	-	-	-	-	0.002^**^	43.2
18-24 years	41	23	7	5	3	3	-	-	14	14	7	3	3	-	-
25-34 years	79	23	28	12	10	6	-	-	25	32	18	4	0	-	-
35-44 years	116	38	40	22	8	8	-	-	32	55	23	4	2	-	-
45-54 years	97	22	31	27	9	8	-	-	21	40	21	12	3	-	-
55-64 years	42	7	13	10	7	5	-	-	15	11	13	3	0	-	-
65 years or above	10	1	2	3	1	3	-	-	0	2	3	4	1	-	-

## Discussion

The results of the current research indicate a significant inverse association between diet quality and cardiovascular risk in South Asian adults. In our study, higher diet quality scores were associated with a lower risk of cardiovascular disease. These findings align with existing data suggesting that nutrient-rich diets containing fibre, antioxidants, and healthy fats are associated with reduced inflammation and lower cardiovascular risk [15]. However, due to the study's cross-sectional design, the relationships identified should be interpreted as associations rather than causal links. Although this study focused primarily on dietary patterns, the potential role of the gut microbiota in mediating these effects remains hypothetical. The link between diet, gut microbiota, and cardiovascular outcomes is inferred rather than directly demonstrated in this study and warrants further exploration in future research through direct microbiome assessments. Prior studies have shown that diets high in fibre enhance the gut microbial synthesis of short-chain fatty acids (SCFAs), which help maintain an anti-inflammatory status, regulate lipid metabolism, and promote endothelial health, all of which are essential pathways for reducing cardiovascular risk [16].

Our findings revealed that the quality of diets was significantly higher in females than in males, suggesting the existence of different gender-related health behaviours. This observation is consistent with prior research indicating that women are healthier, more inclined to follow nutrition recommendations, and more likely to adopt nutritionally acceptable eating patterns [17]. Cultural roles may also influence higher levels of nutritional consciousness and healthy food consumption among females in South Asian households, as women tend to plan meals at home. Conversely, younger men may be more susceptible to the influence of urban fast-food culture, a lack of economic and financial autonomy, and inadequate health literacy. Although females in our sample had significantly higher diet quality scores than males, there was no significant difference in cardiovascular risk scores between the genders. This differs from previous research, indicating that cardiovascular risk increases in ageing women due to hormonal changes, such as the decline in estrogen levels [18]. Such age-related phenomena were not adequately captured in our sample, which is why we did not find significant differences in cardiovascular risk scores.

We found that the diet quality increased with age, and the lowest scores were reported in the youngest age category. This result can be corroborated by past studies showing that older adults are more likely to follow healthier diets compared to young and middle-aged people, and, therefore, calls for specific dietary interventions for younger individuals [19]. We found that younger adults were more likely to score highly on modifiable cardiovascular risk factors, indicating that they were less likely to have healthy life habits. However, this contradicts the existing literature, which suggests that cardiovascular risk is age-related due to the progressive accumulation of metabolic risk factors and tissue ageing. This difference can be explained by the fact that younger adults in our sample may exhibit more unhealthy behaviours, which lead to higher short-term, modifiable risks, even though they result in a smaller age-related clinical burden [20]. These patterns may also be influenced by socioeconomic instability, limited health awareness, and urbanised environments that promote fast food consumption and sedentary lifestyles among younger adults [21].

We found that better diet quality was positively related to greater physical activity, whereas people who were less active perceived their diet as poor. This association has not been adjusted for possible confounding factors, such as age or comorbidities, and should be interpreted as an unadjusted correlation. This is in line with the other literature that showed unhealthy dietary habits tend to cluster with physical inactivity and sedentarism, which shows that lifestyle risk factors associated with obesity and cardiovascular disease are clustered [22]. However, an interesting contradiction emerged in our results: cardiovascular risk scores were highest in individuals who exercised daily. This association persisted even after adjusting for potential confounding variables such as age, gender, and the presence of medical conditions in the multivariable regression analysis. While this may seem counterintuitive, this phenomenon has also been reported in previous studies, which show that vigorous physical exercise is a source of acute cardiovascular risk, especially in people with undiagnosed coronary disease or those with latent heart disorders [23]. This indicates that although regular exercise tends to reduce long-term cardiovascular risk, it can pose a short-term danger in individuals with unobserved coronary problems, which emphasises further research on the complicated topic of exercise and CVD.

In our multivariable regression analysis, high diet quality was a significant predictor of lower cardiovascular risk, consistent with prior research on the potential protective role of nutrition in cardiovascular health. This aligns with literature suggesting that high-quality diets, such as the Mediterranean pattern, rich in bioactive compounds with anti-inflammatory and antioxidant properties, may be associated with lower cardiovascular risk [15]. Gender and age were also essential predictors in our analysis. Unexpectedly, we found that modifiable risk scores were significantly lower among older participants, contrary to classical epidemiological evidence that cardiovascular risk increases among older age groups due to the growing burden of metabolic impairment and tissue wear as we age. This difference can be attributed to healthier food habits, greater medical surveillance, and less participation in risk behaviours among the older adults in our sample, consistent with prior research on age-related improvements in health behaviours [20,19].

In our study, females had slightly lower cardiovascular risk scores than males. This aligns with previous studies, which have shown that women generally engage in healthier lifestyle behaviours, potentially contributing to a reduced cardiovascular risk [24]. Our finding that a higher frequency of physical activity was associated with increased cardiovascular risk aligns with previous research showing that exercise, particularly strenuous activity, may trigger acute cardiovascular events in individuals with undiagnosed heart disease. This suggests the possibility of underlying medical conditions confounding the relationship between activity and risk [23]. Our observation that greater levels of self-rated physical activity are associated with reduced cardiovascular risk is consistent with the literature, which describes positive metabolic and cardiovascular profiles in physically active populations [25]. Lastly, chronic conditions, including hypertension and diabetes, also contributed to greater cardiovascular risk, as reported in previous studies that stated that poor physical health status is a strong predictor of ill outcomes in patients with coronary artery disease and heart failure [26].

The results of the study reveal that physical activity decreases significantly with age; the most active individuals were younger, whereas older participants were relatively less active. This aligns with earlier findings that biological, mechanically driven decreases in motivation to engage in physical activity occur with age, as reported in both animal and human studies [27]. We found that the chronic illnesses, including hypertension and high cholesterol, were considerably driven up in the middle-aged cohorts, but younger adults presented with fewer health concerns. That is in line with the findings of other studies indicating that the burden of chronic diseases tends to increase with age, especially in the elderly who are found in institutions [28].

These results indicate that age and gender-specific interventional dieting is necessary to address South Asian communities. Awareness programs and preventive strategies to promote high dietary quality and reduce modifiable cardiovascular risk factors in young adults, especially males, could be beneficial. In the meantime, maintaining healthier dietary patterns in a geriatric population and strengthening their access to nutritious food resources should also be a target of public health.

Limitations

Although this research offers valuable insights, it is necessary to consider several limitations. First, the study is cross-sectional, so conclusions on causality between diet quality and cardiovascular risks are not possible. The convenience sampling method used in the survey restricts the representativeness of the sample and provides a potential selection bias. There is a possibility that respondents who voluntarily participated in the research had significantly different characteristics (health awareness, socioeconomic status, and/or education) compared to those who did not participate in the study. The results, therefore, have limited generalizable value from the population perspective of the South Asian adult community. This limitation affects the external validity of the study and should be taken into account when interpreting the results.

Additionally, self-reported dietary intake and physical activity can be significantly influenced by recall bias and social desirability bias. In the future, research should employ objective measures, such as biomarkers or activity monitors, to reduce bias. Since self-reported physical activity was uniformly classified only by frequency and not by data regarding activity type and intensity, this could have affected the interpretation of cardiovascular risk scores. Although the gut microbiota composition was indirectly measured through dietary patterns, microbial diversity and specific metabolites were not assessed. Therefore, microbiome-based analyses, particularly 16S rRNA sequencing, could be considered as future additions to the research. Although multivariable regression was performed using age, gender, and medical history, other relevant confounding factors, such as medication use, psychological stress, and genetic predispositions, were not measured or controlled for in this study. Since self-reported physical activity was uniformly classified only by frequency and not by data regarding activity type and intensity, it could have affected the interpretation of cardiovascular risk scores. These unmeasured variables may have influenced cardiovascular risk outcomes and should be considered in future research.

Additionally, while diet-microbiota relationships were inferred through previously validated dietary patterns, no direct measurement of gut microbiota composition or function was conducted. This limits the strength of conclusions regarding microbiome-mediated mechanisms. Future studies should incorporate direct microbiome sequencing to validate these associations more rigorously. Post hoc comparisons were not conducted as well, following the one-way ANOVA analyses, which limits the ability to identify specific group differences within age and physical activity categories. Additionally, the assumption of homogeneity of variance for ANOVA was not formally tested using Levene's test, which may affect the interpretation of group differences. Lastly, although research participants were classified as South Asian, the study was unable to disaggregate the results by ethnic subgroups (e.g., Punjabi, Sindhi, Pathan). Future research must incorporate ethnicity-stratified analyses and investigate the viability and cultural acceptability of microbiota-focused dietary initiatives in a range of South Asian populations.

Future directions

Future studies need to overcome these limitations by considering longitudinal or interventional study designs that can permit causality between dietary patterns, macrobiotic intestinal changes, and cardiovascular consequences. A better understanding of the microbial mechanisms can be gained when direct measurements of gut microbiota are undertaken using metagenomic or 16S rRNA sequencing. By increasing the study group, which will enable a larger and more diverse representation of South Asian communities, the external validity of the findings will be enhanced. Furthermore, objective outcome measures, such as wearable devices, satisfaction, and clinical markers of inflammation, cholesterol, and glucose metabolism, can be assessed to enhance the accuracy of the data. Future work should also consider the inclusion of important confounders such as medication use, psychological stress, and genetic predisposition, which were not accounted for in the present study. Lastly, how targeted dietary interventions that control the gut microbiota, such as the use of prebiotics, probiotics, and dietary personalisation, might be used to reduce cardiovascular risk levels in these individuals should also be investigated in further research. Combining direct microbial assessments with validated measures of dietary quality may offer stronger insights into the gut-heart axis in diverse populations.

## Conclusions

This research provides evidence that diet quality is inversely associated with cardiovascular risk in South Asian adults, suggesting that dietary patterns potentially linked to gut microbiota may contribute to differences in modifiable cardiovascular risk profiles. Individuals with healthier eating behaviours, which are consistent with those previously associated with beneficial intestinal microbial activity, tended to have lower cardiovascular risk ratings. Nevertheless, it did not directly measure the makeup or role of gut microbiota. Thus, the suggested connection remains conjectural and would be worth investigating in a future study through direct testing of the microbiome. Differences in diet quality and risk profiles by age, gender, and ethnicity also underscore the need for targeted interventions in the public health field. Community members in the younger adult age group had notably worse diets and a high risk of CVD.

In contrast, men and women of an older age reported less risk of CVD and possibly healthier eating styles. These results reinforce existing evidence on the potential associative role of fibre-rich, diverse diets in reducing cardiovascular risk, as demonstrated by current literature on nutrition and chronic disease prevention. Ultimately, this study contributes to a substantial body of evidence supporting that microbiota-based dietary interventions may serve as low-cost, scalable, and culturally acceptable approaches to address cardiovascular risk in South Asian populations. These interventions can be implemented as community-based education initiatives that promote affordable and fibre-rich foods, which differ from conventional South Asian dietary patterns and are often found in resource-constrained environments.
